# GPR41 and GPR43 regulate CD8^+^ T cell priming during herpes simplex virus type 1 infection

**DOI:** 10.3389/fimmu.2024.1332588

**Published:** 2024-03-08

**Authors:** Ariane Renita Lee, Kayla Roberta Wilson, Michele Clarke, Sven Engel, David C. Tscharke, Thomas Gebhardt, Sammy Bedoui, Annabell Bachem

**Affiliations:** ^1^ Department of Microbiology and Immunology at the Peter Doherty Institute for Infection and Immunity, University of Melbourne, Parkville, VIC, Australia; ^2^ John Curtin School of Medical Research, The Australian National University, Canberra, ACT, Australia

**Keywords:** CD8^+^ T cells, differentiation, MPEC, GPR41, GPR43, microbiota, SCFA

## Abstract

Naïve CD8^+^ T cells need to undergo a complex and coordinated differentiation program to gain the capacity to control virus infections. This not only involves the acquisition of effector functions, but also regulates the development of a subset of effector CD8^+^ T cells into long-lived and protective memory cells. Microbiota-derived metabolites have recently gained interest for their influence on T cells, but much remains unclear about their role in CD8^+^ T cell differentiation. In this study, we investigated the role of the G protein-coupled receptors (GPR)41 and GPR43 that can bind microbiota-derived short chain fatty acids (SCFAs) in CD8^+^ T cell priming following epicutaneous herpes simplex virus type 1 (HSV-1) infection. We found that HSV-specific CD8^+^ T cells in GPR41/43-deficient mice were impaired in the antigen-elicited production of interferon-gamma (IFN-γ), tumour necrosis factor-alpha (TNF-α), granzyme B and perforin, and failed to differentiate effectively into memory precursors. The defect in controlling HSV-1 at the site of infection could be restored when GPR41 and GPR43 were expressed exclusively by HSV-specific CD8^+^ T cells. Our findings therefore highlight roles for GPR41 and GPR43 in CD8^+^ T cell differentiation, emphasising the importance of metabolite sensing in fine-tuning anti-viral CD8^+^ T cell priming.

## Introduction

Naïve CD8^+^ T cells require priming to fulfil essential roles in the control of intracellular infections and tumours. Priming regulates clonal expansion and the acquisition of cytotoxic effector functions, exemplified by the production of cytokines such as IFN-γ and TNF-α and cytotoxic mediators granzyme B and perforin. Priming signals also determine the trajectory of antigen-stimulated CD8^+^ T cells towards interleukin 7 receptor alpha-chain (IL-7Rα or CD127)-expressing memory precursor effector cells (MPECs) and killer cell lectin-like receptor G1 (KLRG1)-expressing short-lived effector cells (SLECs) (1–3). While terminally differentiated SLECs undergo apoptosis following viral clearance, MPECs persist and give rise to long-lived and protective memory cells (2, 4–6).

T cell differentiation and functionality are regulated by a multitude of factors provided during T cell priming by dendritic cells, including antigen quantity, co-stimulation, T cell help and the cytokine milieu in the microenvironment (7, 8). In recent years it has become apparent that T cell responses are also influenced by the microbiota and their production of metabolites (9–12). Interestingly, microbiota-dependent changes in T cell function extend beyond the gut microbiota-host interface to more distal compartments (13–16). Short-chain fatty acids (SCFAs) for example, that are produced by the microbiota, can be detected in the gut, liver and bloodstream and have been implicated as metabolic and epigenetic regulators of T cells (9, 11, 17, 18). While these effects involve passive diffusion or facilitated transport through cellular membranes, many cells can also sense SCFAs via GPRs expressed on their surface (19–21). Ligation of SCFAs to GPRs reportedly stimulates mitogen-activated protein kinase (MAPK) and extracellular signal-regulated kinase (ERK) cascades (22, 23), which regulate the expression of various immune-related genes (23, 24). While we have started to appreciate that modulations of T cell metabolism impact their differentiation trajectory, in particular their memory potential (18, 25), we understand much less about how sensing of metabolites through GPRs influences CD8^+^ T cell immunity.

The present study was designed to address if SCFA sensing through GPR41 and GPR43 impacts the priming of CD8^+^ T cells following virus infection. We report here that GPR41 and GPR43 expression influences the memory potential, effector molecule production and viral control of CD8^+^ T cells following HSV-1 infection.

## Results

### Impaired CD8^+^ T cell differentiation and cytokine expression in GPR41/43-deficient mice in response to HSV-1 infection

**Figure 1:**
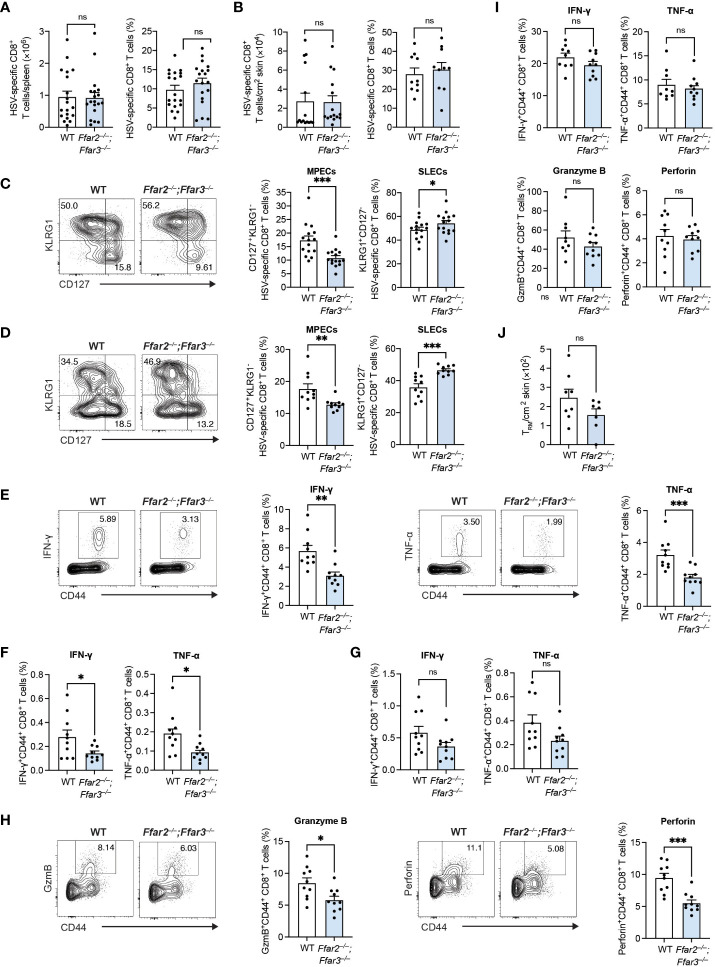
GPR41 and GPR43 promote MPEC differentiation and CD8^+^ T cell function following HSV-1 infection. *Ffar2^–/–^;Ffar3^–/–^* and C57BL/6 wildtype (WT) mice were infected with 1 × 10^6^ plaque-forming units (PFU) HSV-1 epicutaneously. **(A–D)** 9 days after infection, endogenous gB_498-505_-specific CD8^+^ T cells were analysed for absolute numbers and frequencies of total CD8^+^ T cells in the spleen **(A)** and skin **(B)** and proportions of memory precursor effector cells (MPECs) and short-lived effector cells (SLECs) in the spleen **(C)** and skin **(D)**. **(E–I)** Expression of IFN-γ and TNF-α by total CD8^+^ T cells was measured upon a 5-hour *ex vivo* re-stimulation of splenocytes **(E–H)** or cells isolated from the skin **(I)** using gB_498-505_ **(E)**, RR1_822-829_ **(F)** or RR1_982-989_ peptide **(G)** in the presence of brefeldin A. Granzyme B (GzmB) and perforin were analysed without re-stimulation **(H, I)**. **(J)** Absolute numbers of endogenous gB_498-505_-specific CD69^+^CD103^+^ skin T_RM_ were measured 32 days after infection. Data are presented as representative contour plots or bar plots with mean + SEM of n = 8 – 19 mice per group from at least 2 independent experiments. Asterisks indicate statistically significant differences as analysed by Student’s *t*-tests (non-significant (ns) p ≥ 0.05, *p < 0.05, **p < 0.01, ***p < 0.001).

We recently identified a role for GPR41 and GPR43 in memory CD8^+^ T cells following secondary antigen challenge (18), but whether priming of naïve CD8^+^ T cells is influenced by the signalling of these receptors is less clear. To test the impact of GPR41 and GPR43 on CD8^+^ T cell priming following viral infection, we infected C57BL/6 wildtype (WT) mice and mice lacking GPR41 and GPR43 (*Ffar2^–/–^;Ffar3^–/–^*) epicutaneously with HSV-1 and examined the endogenous HSV-specific CD8^+^ T cell response using H2-K^b^-gB_498-505_ tetramers. Splenic gB_498-505_-specific CD8^+^ T cells expanded robustly in both WT and *Ffar2^–/–^;Ffar3^–/–^* mice (**Figure 1A**). In line with this, gB_498-505_-specific CD8^+^ T cells were lodged into the skin of WT and *Ffar2^–/–^;Ffar3^–/–^* mice by day 9 post-infection (**Figure 1B**). Despite similar expansion and skin infiltration of HSV-specific CD8^+^ T cells, we found a 1.7-fold reduction in CD127^+^KLRG1^-^ HSV-specific MPECs in the spleens of *Ffar2^–/–^;Ffar3^–/–^* mice (**Figure 1C**). This corresponded to an increase in KLRG1^+^CD127^-^ HSV-specific SLECs (**Figure 1C**). Similar effects were observed at the site of infection (**Figure 1D**), thus indicating that in the absence of GPR41 and GPR43, the reduction in MPEC differentiation in the spleen impacted T cells in the skin.

We next tested if GPR41 and GPR43 were required for CD8^+^ T cell cytokine production by measuring intracellular IFN-γ and TNF-α following *ex vivo* re-stimulation with gB_498-505_ peptide. Splenic CD8^+^ T cells from GPR41/43-deficient mice produced approximately 1.8-fold less IFN-γ and TNF-α than their WT counterparts (**Figure 1E**). The same trend in cytokine production was observed upon re-stimulation with the subdominant epitopes ribonucleotide reductase 1 (RR1)_822-829_ and RR1_982-989_ (**Figures 1F, G**). We additionally detected significantly lower levels of granzyme B and perforin in *Ffar2^–/–^;Ffar3^–/–^* CD8^+^ T cells compared to WT CD8^+^ T cells (**Figure 1H**).

Despite the impact of GPR41/43 on the phenotype of skin-localized CD8^+^ T cells, cytokine and cytotoxic mediator production was unaffected (**Figure 1I**) and the development of skin resident memory CD8^+^ T cells (T_RM_) was comparable in *Ffar2^–/–^;Ffar3^–/–^* and WT mice (**Figure 1J**). Together these findings revealed an important role for GPR41 and GPR43 in ensuring optimal differentiation and effector molecule production of circulating HSV-specific CD8^+^ T cells *in vivo*.

### The lack of GPR41 and GPR43 expression influences CD8^+^ T cell differentiation independent of antigen load

**Figure 2:**
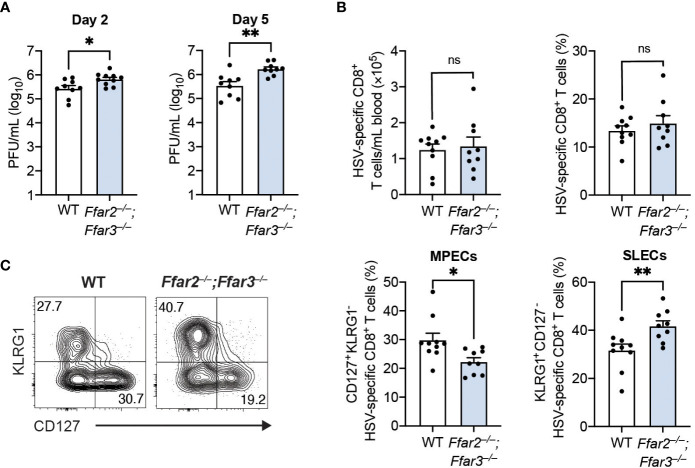
Antigen load does not influence CD8^+^ T cell differentiation in *Ffar2^–/–^;Ffar3^–/–^* mice. *Ffar2^–/–^;Ffar3^–/–^* and WT mice were infected with 1 × 10^6^ PFU HSV-1 epicutaneously **(A)** or with 2 × 10^5^ PFU HSV-1 intravenously **(B, C)**. **(A)** Flank skins were harvested on day 2 and day 5 post-infection for analysis of viral titres using PFU assays. **(B, C)** Analysis of endogenous gB_498-505_-specific CD8^+^ T cells was performed on blood 9 days post-infection. Absolute numbers and frequencies of gB_498-505_-specific CD8^+^ T cells **(B)** as well as MPEC and SLEC proportions **(C)** within total CD8^+^ T cells were measured. Data are presented as representative contour plots or bar graphs with mean + SEM of n = 9 – 10 mice per group from 2 independent experiments. Asterisks indicate statistically significant differences as analysed by Student’s *t*-tests (non-significant (ns) p ≥ 0.05, *p < 0.05, **p < 0.01).

To determine whether these phenotypic and functional differences of cytotoxic CD8^+^ T cells affected control of the virus, we measured HSV-1 titres in the skin. We focused on day 2 and day 5 post-infection, as HSV-1 first undergoes replication at the site of infection (referred to as the primary site) before it spreads to the dorsal root ganglion and re-emerges retroaxonally at the secondary infection site around 3 to 5 days post-infection (26). We found a small but significantly higher viral burden in the skins of GPR41/43-deficient mice compared to WT mice 2 days post-infection (**Figure 2A**). This difference in viral titres between WT and GPR41/43-deficient mice was further increased at 5 days post-infection (**Figure 2A**), demonstrating that GPR41 and GPR43 were required for optimal control at the primary and secondary site of infection.

The higher HSV-1 titres in GPR41/43-deficient mice raised the possibility that antigen availability could be responsible for the differences in CD8^+^ T cell trajectory. To investigate this, we infected mice with HSV-1 intravenously, which results in a non-productive infection (27). Consistent with our findings following epicutaneous infection, expansion of gB_498-505_-specific CD8^+^ T cells in the blood was similar between WT and *Ffar2^–/–^;Ffar3^–/–^* mice (**Figure 2B**), but CD8^+^ T cell differentiation in the latter was significantly skewed away from MPEC differentiation and towards SLEC phenotype (**Figure 2C**). These findings indicate that the defect in effector differentiation of HSV-specific CD8^+^ T cells lacking GPR41 and GPR43 was not an indirect consequence of antigen availability, and instead was a result of SCFA receptor expression.

### GPR41 and GPR43 expression supports memory precursor differentiation through CD8^+^ T cell-extrinsic mechanisms

**Figure 3:**
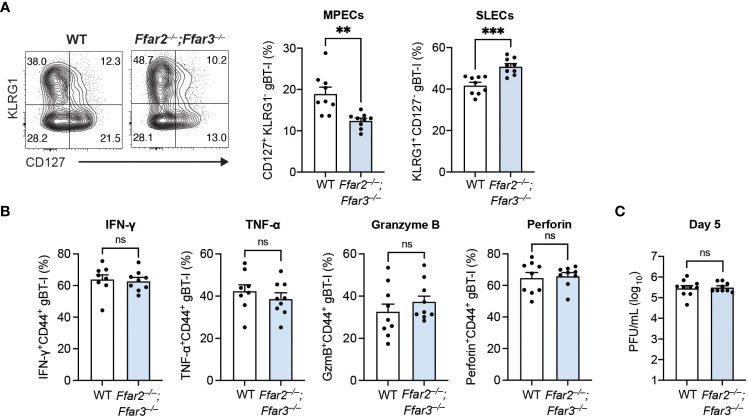
Differentiation into MPECs is hindered in a priming environment deficient in GPR41 and GPR43. *Ffar2^–/–^;Ffar3^–/–^* and WT recipients were transferred 5 × 10^4^ naïve WT gBT-I cells and subsequently epicutaneously infected with 1 × 10^6^ PFU HSV-1. **(A, B)** gBT-I cells were analysed 9 days after infection for SLEC and MPEC proportions in the spleen **(A)**. **(B)** IFN-γ and TNF-α were measured in gBT-I cells from the spleen following re-stimulation with gB_498-505_ peptide for 5 hours in the presence of brefeldin A. GzmB and perforin expression was analysed without re-stimulation. **(C)** Flank skins were harvested 5 days after HSV-1 infection to measure viral titres using PFU assays. Data are presented as representative contour plots or bar graphs with mean + SEM of n = 8 – 10 mice per group from 2 independent experiments. Asterisks indicate statistically significant differences as analysed by Student’s *t*-tests (non-significant (ns) p ≥ 0.05, **p < 0.01, and ***p < 0.001).

Having identified a reduction in MPEC differentiation and function with global deficiencies in GPR41 and GPR43, we next sought to delineate whether this receptor signalling requirement was CD8^+^ T cell-intrinsic. For this, we adoptively transferred naïve transgenic CD8^+^ T cells carrying T cell receptors against the HSV-derived, immunodominant gB_498-505_ epitope (gBT-I cells) into recipients deficient or sufficient for GPR41 and GPR43 one day prior to epicutaneous HSV-1 infection. This allowed us to test the isolated role of GPR41 and GPR43 on HSV-specific CD8^+^ T cells. Our analysis of the gBT-I cell differentiation trajectory uncovered that the GPR41/43-competent gBT-I population in *Ffar2^–/–^;Ffar3^–/–^* recipients contained a lower proportion of MPECs than in WT recipients (**Figure 3A**). Consistent with this, the relative prevalence of GPR41/43-competent SLECs was greater in *Ffar2^–/–^;Ffar3^–/–^* recipients (**Figure 3A**). The influence of GPR41 and GPR43 expression on MPEC/SLEC differentiation during priming can thus be attributed to CD8^+^ T cell-extrinsic mechanisms.

Interestingly, we observed similar IFN-γ, TNF-α, granzyme B and perforin expression by GPR41/43-competent gBT-I cells from WT and *Ffar2^–/–^;Ffar3^–/–^* hosts (**Figure 3B**), indicating that GPR41 and GPR43 expression on CD8^+^ T cells was sufficient for optimal effector function. Of note, viral titres at the secondary infection site of WT and GPR41/43-deficient hosts were similar when GPR41/43-competent gBT-I cells were transferred (**Figure 3C**), demonstrating that GPR41/43 expression on HSV-specific CD8^+^ T cells alone was sufficient to ensure optimal control of the virus infection.

## Discussion

We report a previously unappreciated role for the SCFA receptors GPR41 and GPR43 in impacting the differentiation trajectory of virus-specific CD8^+^ T cells following HSV-1 infection. GPR41/43 expression was required for optimal memory precursor differentiation and effector molecule production, but the receptors were dispensable for the regulation of the magnitude of the response. The altered differentiation of HSV-specific CD8^+^ T cells was associated with a significant increase of viral titres at the site of infection. This defect could be restored by transferring GPR41/43-competent HSV-specific CD8^+^ T cells, suggesting that the defect in control was at least in part due to optimal IFN-γ, TNF-α, granzyme B and perforin expression by virus-specific CD8^+^ T cells requiring GPR-mediated SCFA signals.

Memory differentiation is vital to maintain a population of antigen-experienced CD8^+^ T cells to confer protection. Several factors, including the strength of antigenic stimulation, co-stimulation, CD4^+^ T cell help and inflammatory signals, influence this T cell trajectory (7, 8). Additionally, the memory potential of T cells can be impacted by alterations in their metabolism, for example by drugs, such as rapamycin and metformin, or microbiota-derived SCFAs (18, 25, 28) but our previous work suggests that SCFA-induced metabolic modulations can be independent of GPR41/43 (18). In contrast to these direct metabolic interventions, the role of metabolite sensing by GPRs in T cell differentiation is not as clear. Our findings highlight that SCFA sensing through GPRs can impact T cell differentiation and cytokine production. The requirement for GPR41 and/or GPR43 for optimal CD8^+^ T cell differentiation towards MPECs and effector molecule expression is in line with a role for SCFAs and GPR41/43 sensing in differentiation and functionality of antigen-experienced CD8^+^ T cells (18) and cytokine production following influenza infection (29). Importantly, these findings highlight that SCFAs cannot only impact T cell differentiation by incorporation into the tricarboxylic acid cycle enhancing oxidative phosphorylation (18), but additionally through receptor-mediated sensing. GPR41 and GPR43 signalling initiates MAPK and ERK cascades (22, 23) that reportedly regulate T cell cytokine production (30–33). Further work will be required to resolve these GPR41/43-induced signalling cascades in CD8^+^ T cells upstream of genes encoding these effector molecules. Also, the extrinsic source impacting the differentiation of CD8^+^ T cells is unresolved. Given the high expression of *Ffar2* in dendritic cells, it is tempting to speculate that CD8^+^ T cell priming could be directly impacted (34). The difference in viral load 2 days after infection further suggests that the expression of GPR41 and GPR43 by cells outside of the adaptive immune response contributes to viral control.

GPR41/43 are important for the recall response of circulatory memory CD8^+^ T cells (18), whereas T_RM_ development was not affected. Possible explanations include low concentrations of microbiota-derived metabolites in the skin, a preference of T_RM_ for other metabolites, or perhaps additional metabolite-sensing receptors expressed by T_RM_. This is consistent with the findings from Pan and colleagues who showed that T_RM_ can use fatty acid-binding protein (FABP)4 and FABP5 to take up fatty acids in the skin (35). It is therefore tempting to speculate that SCFA sensing by GPRs provides the host with the capacity to integrate microbiome- and stress-related changes into circulatory CD8^+^ T cell responses. In line with this hypothesis, bacterial infections can alter the levels of the SCFA acetate, and this has been shown to influence cytokine production by CD8^+^ T cells (36, 37). Whether epicutaneous HSV-1 infection modulates SCFA levels as well as the source of SCFA production following HSV-1 infection remains to be determined. However, intranasal infection of mice with HSV-1 alters the gut microbiome (38), indicating a potential for HSV-1 to impact microbiota-derived SCFA levels.

The emerging view of the immunomodulatory potential of SCFAs makes them and their receptors potential therapeutic targets for inflammatory conditions, infections, and cancer. Indeed, synthetic compounds with GPR43 agonistic properties reportedly attenuate dextran sulphate sodium-induced colitis in mice (39). By identifying the impact of GPR41/43 expression on CD8^+^ T cell differentiation and cytokine responses, we have provided a foundation for future studies designed to manipulate these GPRs for the purpose of long-term and protective T cell memory.

## Methods

### Mice

Female C57BL/6 (WT), *Ffar2^–/–^;Ffar3^–/–^* and gBT-I × WT.CD45.1 (gBT-I.CD45.1) mice were bred and maintained in specific pathogen-free conditions in the Bioresources Facility in the Department of Microbiology and Immunology at the University of Melbourne. gBT-I.CD45.1 mice express transgenic T-cell receptors that recognise the HSV glycoprotein B-derived epitope gB_498–505_ in the context of major histocompatibility complex class I presentation (40). The congenic marker CD45.1 was used to differentiate gBT-I cells from the endogenous cells of recipient mice.

### Herpes simplex virus type 1 infection and determination of plaque forming units

The KOS strain of HSV-1 was propagated and titrated in Vero cells (CSL Parkville) and mice were epicutaneously infected as previously described (26). In short, mice were anesthetised with ketamine (100 mg/kg bodyweight) and xylazine (15 mg/kg bodyweight) via intraperitoneal injection. The left flank of the mouse was shaved, followed by depilation using Veet hair removal cream. The skin was lightly abraded, and 1 × 10^6^ plaque forming units (PFU) of HSV-1 were applied to the site. Second skin (Op-Site Flexigrid) was used to cover the site, and mice were bandaged with Micropore and Transpore surgical tape. Bandages were removed after 2 days. For intravenous injections, 2 × 10^5^ PFU of HSV-1 were injected in 200 μL phosphate buffered saline (PBS).

Viral titres at the site of infection were determined as previously described (26). In summary, 2 cm × 0.5 cm skins were homogenised and incubated for 1 hour in wells containing a confluent monolayer of Vero cells. A 1% agarose layer was added to prevent spread of the infection. Plates were incubated in a 37°C incubator for 4 days. Vero cells were fixed with formalin and stained with 0.1% Toluidine blue to enumerate plaques.

### Adoptive transfer of gBT-I.CD45.1 cells

Skin-draining lymph nodes were harvested from naïve gBT-I.CD45.1 mice and single cell suspensions prepared by meshing organs through a 70 μM sieve. The numbers of gBT-I.CD45.1 cells were determined using trypan blue exclusion and flow cytometry. 5 × 10^4^ naïve gBT-I.CD45.1 cells were transferred into naïve recipients in 200 μL PBS intravenously.

### Preparation of single cell suspensions

Blood was collected from the submandibular vein using a heparinised capillary tube. Red blood cell lysis was performed by treating 50 μL blood twice with 1 mL of red blood cell lysis buffer (1.56 M ammonium chloride, 1.27 mM ethylenediaminetetraacetic acid, 0.119 M sodium bicarbonate in Baxter water) followed by a wash in FACS buffer.

For the harvest of all other organs, mice were sacrificed by carbon dioxide inhalation. Single cell splenic suspensions were generated by pressing spleens through 70 μm filters.

For endogenous T cell analysis of the skin, 2 cm × 0.5 cm skin surrounding the primary and secondary infection site was harvested directly into liberase solution (0.5 mg/mL Liberase TL Research Grade and 10 μg/mL DNase I in Hank’s balanced salt solution), followed by incubation in a 37°C water bath for 30 minutes. The epidermal and dermal layers were separated, finely cut and incubated in liberase solution at 37°C for 60 minutes. Samples were homogenised by resuspending with a transfer pipette, then filtered using nylon meshes.

### Flow cytometry

Single cell suspensions were stained with fluorescent-labelled antibodies in PBS supplemented with 10% w/v bovine serum albumin and 5 mM ethylenediaminetetraacetic acid for 20 minutes. The BD Cytofix/Cytoperm kit was used for intracellular cytokine stains following the manufacturer’s protocol. H2-K^b^-gB_498-505_ tetramers were used to identify HSV-specific CD8^+^ T cells as published previously (26). The following antibodies from BD Biosciences were used: anti-CD8α (Clone 53-6.7), anti-CD44 (IM7), anti-CD45.1 (A20), anti-CD45.2 (104), anti-CD69 (H1.2F3), anti-CD103 (M290), anti-CD127 (A7R34), anti-granzyme B (GB11) anti-IFN-γ (XMG1.2), anti-KLRG1 (2F1), anti-perforin (S16009A), anti-TNF-α (MP6-XT22), and anti-Vα-2 (B20.1). Dead cells were excluded using propidium iodide or LIVE/DEAD fixable dead cell stain kit (ThermoFisher) according to the manufacturer’s description. HSV-specific CD8^+^ T cells were re-stimulated *ex vivo* by incubation with brefeldin A (Sigma Aldrich) and either 1 μg/mL gB_498-505_ (GenScript), RR1_822-829_ (GL Biochem) or RR1_982-989_ peptide (GL Biochem) for 5 hours at 37°C (41–43). Unstimulated samples were incubated with brefeldin A only. Cell numbers were calculated using Sphero Blank Calibration Particles (BD PharMingen). Samples were measured using a BD LSRFortessa. FlowJo 10 software was used for analysis.

### Statistical analysis

Statistical analysis was performed using GraphPad Prism 9. P values were calculated using Student’s *t*-tests, as indicated in figure legends. Statistical significance was achieved when p values were less than 0.05. Significance values were denoted as non-significant (ns) p ≥ 0.05, * p < 0.05, ** p < 0.01, *** p < 0.001.

## Data availability statement

The original contributions presented in the study are included in the article/supplementary material. Further inquiries can be directed to the corresponding author.

## Ethics statement

The animal study was approved by The University of Melbourne Animal Ethics Committee. The study was conducted in accordance with the local legislation and institutional requirements.

## Author contributions

AL: Conceptualization, Data curation, Formal analysis, Investigation, Methodology, Visualization, Writing – original draft, Writing – review & editing. KW: Formal analysis, Investigation, Writing – review & editing. MC: Investigation, Methodology, Writing – review & editing. SE: Formal analysis, Investigation, Writing – review & editing. DT: Methodology, Writing – review & editing. TG: Conceptualization, Writing – review & editing. SB: Conceptualization, Data curation, Funding acquisition, Supervision, Writing – original draft, Writing – review & editing. AB: Conceptualization, Data curation, Investigation, Methodology, Supervision, Writing – original draft, Writing – review & editing.
